# Influencing Factors and Countermeasures of the Health of Residents in the City Clusters along the Middle Reaches of the Yangtze River

**DOI:** 10.3390/healthcare8020093

**Published:** 2020-04-10

**Authors:** Qizhong Deng, Yuyan Yi, Keming Yu

**Affiliations:** 1 School of Business, Hunan University of Science and Technology, Xiangtan 411201, China; yiyuyan2000208@163.com; 2 Department of Mathematics, Brunel University London, London UB83PH, UK; keming.yu@brunel.ac.uk

**Keywords:** the city clusters along the middle reaches of the Yangtze river, health of residents, medical level, production function for health

## Abstract

This paper introduces several factors, namely, environmental pollution, medical level and environmental governance, into the Grossman’s production function for health. Then, an empirical analysis was conducted based on the 2004–2016 panel data of the city clusters along the middle reaches of the Yangtze River. Through the analysis, the author evaluated and compared how different factors affect the health of residents in the three city clusters: Changsha-Zhuzhou-Xiangtan (CZT) city cluster, Wuhan city cluster and circum-Poyang Lake (CPL) city cluster. The results show that: (1) In all three city clusters, economic growth can effectively improve the health of residents, and environmental pollution is also a key influencing factor of the health of residents. (2) Medical level has a close correlation with the health of residents. In the CZT city cluster, the medical level is positively correlated with the health of residents; in the CPL city cluster, the correlation is negative and takes the shape of an inverted U in the long run. (3) In all three city clusters, the environmental governance has an inverted U-shape correlation with the health of residents, indicating that environmental governance is not enough to promote the health of residents. Finally, several countermeasures were put forward to enhance the health of residents in the study area. The research findings shed new light on policymaking for the health of residents.

## 1. Introduction

Since the reform and opening up, China has witnessed a sustained rapid growth of economy. However, the extensive mode of economic growth poses severe challenges to the energy and environment. The resulting health problems have become a major social concern. In May 1990, the United Nations Development Program (UNDP) released the first Human Development Report. From then on, the UNDP has reiterated that human wellbeing is the ultimate goal of development. An important aspect of human wellbeing lies in health, an important element of human capital and a direct impetus for social productivity. Therefore, promoting the health of residents is an important means to improve their quality of life and a long-term strategy for coordinated development between the economy and society. 

The city clusters along the middle reaches of the Yangtze River are located at the center of the Yangtze River Economic Belt. These city clusters occupy a very important position in China’s pursuit of green coordinated development. Currently, these city clusters are striving to enhance the greenness and sustainability of its development mode. Against this backdrop, it is of great theoretical and practical significance to improve the health of local residents, while coordinating the harmonious development of the economy and environment.

The health of residents has long been a research hotspot. The paper [1] was the first to introduce the health factor to the utility function and created the model of health demand, laying the theoretical basis for health research. Since then, various methods have been adopted to explore and compare how different factors affect the health of residents, ranging from the analytical method, media analysis method, survey analysis to metric method [2,3,4,5]. The relevant studies mainly concentrate on three aspects, namely, the economic issue, environmental issue and public service.

On the economic issue, most scholars agree that, in the long run, the health of residents is promoted by economic growth [6,7] and suppressed by income inequality [8,9], whether on a microscale or macroscale. However, some scholars held the opposite view: economic level has a negative correlation with the health of residents, without considering the effect of time [10,11]. Some other scholars believed that the relationship between economic growth and the health of residents changes cyclically [12].

On the environmental issue, the academia has arrived at a relatively consistent view: environmental pollution has a significant negative effect on the health of residents. The environmental degradation triggers personal health problems on the microscale [13] and also pushes up mortality and morbidity on the macroscale [14]. Some scholars also evaluated the health risks brought on by environmental pollutions to infants [15], seniors [16] and rural populations [17].

On public health, there is a growing interest in the influence of education level [18], medical level [19], public health expenditure [20] and transfer payment [21] on the health of residents. However, the relevant studies have drawn different conclusions [22,23].

Despite their sheer number, the existing studies on the health of residents have several defects. First, there is little reported that measures the impacts of multiple factors on the health of residents. Second, most scholars discussed the degree of influence of varied factors on the health of residents only through a time series analysis, and the results may have erroneous due to the lack of data samples. Third, most studies focused on the national or provincial level, failing to consider the difference between city clusters in economic development.

To overcome these defects, this paper introduces several factors, namely, environmental pollution, medical level and environmental governance, to a Grossman model of health demand. Then, the 2004~2016 panel data of the city clusters along the middle reaches of the Yangtze River were subjected to unit root test, cointegration test and regression analysis, aiming to verify if there was a stable equilibrium between the variables. In addition, the authors discussed the impacts of the above factors on the health of residents. Finally, several feasible suggestions were put forward for incentive policies on the green, coordinated and harmonious development of the city clusters along the middle reaches of the Yangtze River.

The remainder of this paper is organized as follows: Section 2 explains the research methods, model construction and data sources; Section 3 performs the empirical analysis and interprets the results and Section 4 puts forward the conclusions and suggestions.

## 2. Methodology

### 2.1. Research Methods

#### 2.1.1. Unit Root Test of Panel Data

As the numerical characteristics of a nonstationary time series change with time, the regression model built directly with these data will have a pseudo-regression phenomenon. Therefore, before regression of the panel data, a unit root test is needed. The following Auto-regressive (1) process is considered for the panel data:

(1) $$Y_{it}=\rho_{i}Y_{it-1}+X_{i}\delta_{i}+\mu_{it}\;(i=1,2,\cdots ,N,\;t=1,2,\cdots ,T_{i})$$

In Equation (1), $X_{it}$ represents the exogenous variable vector in the model, including the fixed effect and time trend of each cross-section. N represents the number of members of the section. $T_{i}$ represents the observation period of the *i*th section member. The random error terms $\mu_{it}$ satisfy the hypothesis of independent and identical distribution. $\rho_{i}$ is the autoregressive coefficient; if $\left|\rho_{i}\right|<1$, the corresponding sequence $Y_{it}$ is a stationary sequence. If $\left|\rho_{i}\right|=1$, the corresponding sequence $Y_{it}$ is a nonstationary sequence.

Depending on the different limits of the argument $\rho_{i}$ in Equation (1), the unit root test methods of the panel data fall into two categories. The typical methods of the first category include the Levin–Lin–Chu (LLC) test, the Breitung test and the Hadri test. These types of methods assume that the cross-section sequences of the panel data have the same unit root process, i.e., $\rho_{i}=\rho \left(i=1,2,\ldots ,N\right)$.

The methods of the second category allow the cross-section sequences of the panel data to have different unit root processes. In other words, parameter $\rho_{i}$ can change across each cross-section. The popular methods in this category include the Im-Pesaran-Shin (IPS) test, Augmented Dickey-Fuller (ADF)-Fisher test and Phillips-Perron (PP)-Fisher test.

In this paper, the commonly used LLC test and ADF-Fisher test are conducted in three modes: intercept term only, intercept term and trend term and no intercept term or trend term.

#### 2.1.2. Cointegration Test of Panel Data

The panel data can be subjected to a cointegration test if all variables are integrated in the same order. There are roughly two types of cointegration tests. One is the cointegration tests based on the Engle-Granger twostep method, such as the Pedroni test and Kao test. The other generally refers to the Fisher test of panel cointegration based on the Johansen cointegration test.

This paper verifies the cointegration relationship of the panel data through the Kao test based on the homogenous panel and the Pedroni test based on the heterogeneous panel. Specifically, the Kao test covers the Dickey-Fuller (DF) test and the ADF test, assuming that the variables have no cointegration relationship. The Pedroni test constructs 7 statistics based on the regression residuals of the cointegration equation and uses them to evaluate the cointegration between panel variables.

### 2.2. Theory and Model Construction of Health Requirement

#### 2.2.1. Health Demand Theory

Grossman introduced the health utility function on the basis of Becker’s family production function, constructed the health demand theory model and proposed the basic concept of health capital. The basic framework of the model is as follows.

Suppose that the utility function of a representative consumer is:

(2) $$U=U\left(\varphi_{t}H_{t},Z_{t}\right),t=0,1,\cdots ,n$$

Among them, $H_{t}$ is the health capital stock of phase t. $\varphi_{t}H_{t}$ is the health of the consumption in phase t. $Z_{t}$ is the other goods besides those health-consumed in phase t. The initial healthy capital stock $H_{0}$ is exogenous. The $H_{t}$ of each subsequent period is an endogenous variable, which is selected by the consumer. The consumer lifespan n is also endogenous. The increment of the health capital is:

(3) $$H_{t+1}-H_{t}=I_{t}-\delta_{t}H_{t}$$

Among them, $I_{t}$ is the healthy capital investment in the period t. $\delta_{t}$ is the depreciation rate, which is also assumed to be exogenous and varies with age. $I_{t}\;\mathrm{and}\;Z_{t}$ are determined by the following functions:

(4) $$I_{t}=I_{t}\left(M_{t},TH_{t};E\right)$$

(5) $$Z_{t}=Z_{t}\left(X_{t},T_{t};E\right)$$

Among them, $M_{t}$ is a set of goods that can be purchased and will be used as input to produce $I_{t}$. $TH_{t}$ is used to improve the health time. $X_{t}$ is a general consumable that can be purchased. $T_{t}$ is the time spent producing $Z_{t},$ and all four are endogenous. E is for the exogenous human capital in addition to health, so the budget constraints consumers face are:

(6) $$\overset{n}{\underset{t=0}{\sum }}\frac{P_{t}M_{t}+Q_{t}X_{t}}{{\left(1+r\right)}^{t}}=\overset{n}{\underset{t=0}{\sum }}\frac{W_{t}TW_{t}}{{\left(1+r\right)}^{t}}+A_{0}$$

Among them,$\;P_{t}$ and $Q_{t}$ are the prices of $M_{t}$ and $X_{t},$ respectively. $W_{t}$ is the salary rate. $TW_{t}$ is the working time. $A_{0}$ is the initial wealth, and r is the interest rate. In addition to budget constraints, consumers face time constraints. The total time available in each period is ω and must be used up in the current period, so the time constraint is:

(7) $$TW_{t}+TH_{t}+T_{t}+TL_{t}=\omega$$

$TL_{t}$ is the time lost due to poor health. The above equations constitute the health demand model of consumers, whose goal is to maximize the total utility under budget and time constraints.

On this basis, more and more scholars supplement and improve the model and gradually form a more mature theory of health demand. The main contents are as follows: (1) consumers’ health demand is health itself, and medical and health services are merely derived demands generated in the process of consumers’ investments in health. (2) Health has dual attributes. On the one hand, it is regarded as a consumer product and directly enters into the utility function of individuals. Health, on the other hand, as an investment reduces the total amount of time available for market or nonmarket activities due to illness. (3) Consumers have a certain initial health capital stock. In order to make up for health depreciation, they will make health investments to produce health to continuously improve their total utility, such as purchasing medical services and taking physical exercise. (4) The higher the consumer’s income, the higher the rate of return on staying healthy. At the same time, education is also one of the important factors affecting health. The higher the education level is, the lower the cost of health will be.

#### 2.2.2. Model Construction

Our model was constructed based on the Grossman’s production function for health. Drawing on the research results of [14,19,24], the economic growth was selected as the input of the production function for health, the environmental pollution was taken as the mediating variable, while the medical level and environmental governance were adopted as exogenous explanatory variables. On this basis, the following model was established:

(8) $$Health_{it}=c_{it}+\beta_{1}lngdpgr_{it}+\beta_{2}lnenvp_{it}+\beta_{3}lnhosp_{it}+\beta_{4}envm_{it}+\mu_{it}$$

where $haelth_{it}$ is the health level of residents in city cluster *i* at year *t*; $lngdpgr_{it}$ and $lnenvp_{it}$ are the economic growth and environmental pollution of city cluster *i* at year *t*, respectively; $lnhosp_{it}$ and $envm_{it}$ are two control variables representing the medical level and environmental governance, respectively; $\beta_{1}$, $\beta_{2}$, $\beta_{3}$ and $\beta_{4}$ are estimated parameters reflecting the degree of influence of each variable on the health of residents and $\mu_{it}$ is a random disturbance term of each cross-section. To alleviate problems like data collinearity and heteroscedasticity, all explanatory variables other than environmental governance were expressed in logarithmic form, making the sample data more stable.

### 2.3. Variable Selection

The explained variable is the health of residents in each city cluster. Based on data availability and comparability, referring to the World Health Organization’s (WHO’s) definition of health and the production functions for health proposed by [19,21], this paper decides to measure the health of residents in each city cluster by the mortality rate. At the same time, this is also the indicator commonly used in the current health production function in macro research [14,19]. This is a contrary index: the higher the mortality rate, the poorer the health of the residents.

The main explanatory variables include economic growth and environmental pollution. Based on data representation, the regional GDP growth rate was selected to measure the economic growth. This index can accurately reflect how fast the economy grows in a region and has been widely used. Since different regions have different economic aggregates, different economic structures and large differences in the number of employees; the GDP growth rate, as one of the important reference indicators for the horizontal and vertical comparisons of economic levels, can effectively describe the macro development of a regional economy. 

There is no unified standard for measuring environmental pollution at home and abroad. Therefore, the environmental pollution was represented by the integrated value of three indices: industrial wastewater discharge, industrial SO_2_ emissions and industrial soot emissions. Considering that industry is still the main driving force supporting the economic growth of city clusters in the middle reaches of the Yangtze River, and industrial emissions have become the main source of environmental pollution in the process of industrial development, these three indicators are the variables that most directly affect the health status of residents. In addition, several issues were considered before selecting the three factors: the residents are constantly exposed to external water bodies and the air; water and air pollution have direct impacts on the health of residents. However, there are strong overflow benefits and exogenous determinacy in the water and atmosphere environments, which is conducive to reducing the endogenous problem of data. In addition, the data of the three kinds of pollution emissions are more comprehensive and objective than other measurement indicators, which can effectively avoid the lack of macro data in the use and is also a common index for many scholars to conduct such research. 

Other control variables include the medical level and environmental governance. The former is expressed by the number of health institutions. The government’s increased investment in medical resources can strengthen the prevention and treatment of diseases and improve the medical level, so as to significantly improve the health status of residents. The number of health institutions, as the infrastructure, to some extent reflects the regional financial investment in medical undertakings, which is often regarded as an important indicator to evaluate the level of medical resources. The city cluster in the middle reaches of the Yangtze River is located in the central region, and the economic development degree of each city is quite different, which makes the financial investment in medical and health undertakings of each region have a big difference. Therefore, the number of medical institutions was used to represent the medical level of each city group. 

The environmental governance is measured by the average of the municipal sewage treatment rate, the comprehensive utilization rate of industrial solid waste and the harmless treatment rate of household garbage. The treatment of municipal sewage, industrial solid waste and domestic waste is the main measure of environmental control in the research area. The data of these three indicators, as the main evaluation of the monitoring of ecological protection and construction in China, comprehensively reflect the strength of urban environmental governance. Meanwhile, in view of the openness and availability of data, the mean value of these three indicators was chosen to describe the level of environmental governance in this region.

### 2.4. Objects and Data Sources

Our empirical analysis targets the 2004–2016 panel data on the city clusters along the middle reaches of the Yangtze River, which involves 28 cities in the Changsha-Zhuzhou-Xiangtan (CZT) city cluster, Wuhan city cluster and circum-Poyang Lake (CPL) city cluster. The CZT city cluster, Wuhan city cluster and the CPL city cluster contain 8, 10 and 10 cities, respectively.

The basic data were collected from *China City Statistical Yearbook*, *China Statistical Yearbook for Regional Economy*, and the yearbooks and socioeconomic development statistical bulletins released by relevant provinces and municipalities. The missing data were padded based on the mean growth rate of the corresponding index in the study period, with the initial year of the available data as the base period.

The statistical descriptive results of each variable are shown in Table 1. It can be found that the maximum mean of the mortality rate occurs in the urban agglomerations around the Changsha-Zhuzhou-Xiangtan (CZT) city cluster, and the minimum mean in the urban agglomerations around the Wuhan city cluster. The largest mean of the GDP growth rate appeared in the circum-Poyang Lake (CPL) city cluster, while the smallest mean was in the Wuhan city cluster. At the same time, the maximum mean values of environmental pollution, medical treatment level and environmental treatment are all in the urban agglomeration around the Changsha-Zhuzhou-Xiangtan (CZT) city cluster, and the minimum mean values of medical treatment level and environmental treatment are in the Wuhan city cluster. Combined with the data of population mortality, it can be speculated that the above variables may all be important factors affecting the health status of residents in the region.

## 3. Empirical Analysis

### 3.1. Unit Root Test of Panel Data

Before the regression analysis, the panel data must receive a unit root test to avoid spurious regression. In this paper, the panel data are inspected by the LLC test and the ADF-Fisher test using EViews 8.0. The test results show that most data in the original sequences cannot reject the null hypothesis, indicating that the sequences are nonstationary ones with unit roots. After first-order difference processing, all variables passed the significance test at the 1% level and can reject the null hypothesis. Hence, the processed sequences are integrated of the first order.

### 3.2. Cointegration Test of Panel Data

Since the sequences of our model are integrated of the first order, the cointegration of the panel data was examined through the Pedroni test and Kao test. The cointegration was mainly judged by the ADF statistic in the Kao test and the Panel PP, Panel ADF, Group PP and Group ADF statistics of the Pedroni test. According to the test results in Table 2, all variables passed the significance test at the 10% level in the Pedroni test; in the Kao test, the *p*-value of the CPL city cluster was significant at the 5% level, while that of any other city cluster was significant at the 1% level. Therefore, the health of residents in each city cluster must have a cointegration relationship with economic growth, environmental pollution, medical level and environmental governance.

### 3.3. Regression Analysis of Panel Data

Before modeling the panel data, the Hausman test was carried out to detect the existence of the sequence correlation and heteroscedasticity. Based on the test results, the author decided to adopt the fixed effect model. In addition, the panel-corrected standard errors (PCSE) method was employed to conduct seemingly unrelated regressions of the panel data on the CZT city cluster, Wuhan city cluster and the CPL city cluster, respectively. Since the number of cross-sections is greater than the number of time series, the panel data of the city clusters along the middle reaches of the Yangtze River were regressed after weighting each cross-section. Table 3 lists the influence of each variable on the health of residents.

As shown in Table 3, the health of residents in each city cluster was significantly affected by the economic growth and environmental pollution, before the addition of the exogenous explanatory variables. 

When the model only contains one explanatory variable: economic growth, the economic growth in each city cluster could effectively promote the health of local residents. The faster the economic growth, the better the health of the local residents.

Once the environmental pollution was introduced to the regression equation, the correlation between economic growth and mortality rate remained negative in all city clusters, but the absolute value of the coefficient of the impact from economic growth on the mortality rate decreased by varied degrees. The absolute value of the impact coefficient dropped from 0.68 to 0.64 in the CZT city cluster, from 0.61 to 0.52 in the Wuhan city cluster, from 0.21 to 0.17 in the CPL city cluster and from 0.43 to 0.34 in city clusters along the middle reaches of the Yangtze River. Thus, environmental pollution exerted the most prominent mediating effect on the relationship between economic growth and mortality rate in the Wuhan city cluster.

The above results show that the increase of environmental pollution not only reduces the health utility of residents but also lowers economic benefits and raises economic costs.

As shown in Table 4, after all the other control variables had been added, the correlation between the economic growth and mortality rate remained negative in all city clusters; this variable passed the significance test at the 1% level. The results demonstrate that economic growth can effectively suppress the mortality rate and enhance the health of residents.

Besides, environmental pollution significantly weakened the health of residents in the Wuhan city cluster, the CPL city cluster and city clusters along the middle reaches of the Yangtze River. Hence, growing environmental pollution poses a greater risk to the health of residents; i.e., the environment quality of a region is closely related to the health of local residents.

In addition, the medical level in the CZT city cluster showed a negative correlation with the mortality rate and passed the significance test at the 5% level. This means the improving medical level has a positive impact on the health of the local residents. Note that the medical level in the CPL city cluster had a positive correlation with the mortality rate, indicating that the local medical resources are too limited to enhance the health of local residents.

Furthermore, the environmental governance did not improve the health of residents, as expected in any of the three city clusters. The coefficient of the impact from environmental governance on the mortality rate was positive in all the three city clusters and passed the significance test. Therefore, the investment on environmental governance in city clusters along the middle reaches of the Yangtze River has not suppressed the health risks of local residents but exerted a certain negative effect instead.

However, it is counterintuitive that the mortality rate remains positively correlated with the medical level and environmental governance in the long term. The positive correlations could be attributable to the fact that the medical level and environmental governance have not reached the threshold to effectively reduce health risks. 

Therefore, this paper adds the quadratic term of the logarithm of the medical level to the original model for the CPL city cluster (Model 15). After the addition, the impact coefficients of all variables were still significant, and their signs remained unchanged. The coefficient of the quadratic term was significantly negative at the 10% level, revealing the inverted U-shape correlation between the medical level and mortality rate. It indicates that only when the medical level reaches an inflection point can the health level of residents be truly and effectively promoted. It also proves that the current medical infrastructure is not complete, and the medical investment is insufficient, which makes the medical level of the region unable to reduce the damage to the health of residents caused by environmental pollution and other factors. In addition, the medical input cost is large, and the low level of public medical input makes it difficult to bring about an obvious positive effect in a short period of time.

In addition, the quadratic term of the logarithm of environmental governance was also introduced to the model for each city cluster. The results show that the coefficients of all first-order terms in each model were still significant, and their signs remained unchanged. The coefficient of the quadratic term was significantly negative, showing an inverted U-shape correlation between the environmental governance and the health of residents. This means the environmental governance increases with the mortality rate before reaching the inflection point of the U-shape curve and will suppress the mortality rate after passing that point. The limited effect of the environmental governance mainly comes from the lack of relevant efforts, the insufficiency of pollution control and the relaxed monitoring standards.

## 4. Conclusions

Based on the 2004–2016 panel data of the city clusters along the middle reaches of the Yangtze River, this paper empirically analyzes the impacts of economic growth, environmental pollution, medical level and environmental governance on the health of residents in the CZT city cluster, Wuhan city cluster and the CPL city cluster. The main conclusions are as follows:

(1) There is a long-term equilibrium relationship between the health of residents in each city cluster with economic growth and environmental pollution. Both economic growth and environmental pollution have important impacts on the health of residents. The medical level of each city cluster is also closely related to the health of local residents. However, the environmental governance is insufficient in the city clusters, failing to promote the health of residents in the study area.
(2) The economic growth of city clusters along the middle reaches of the Yangtze River has a significant positive influence on the health of residents. The sustained and steady development of the economy can bring good health benefits. Environmental pollution mediates the positive impacts of the economy on the health of residents. It is a key negative influencing factor of the health of residents.
(3) In the CZT city cluster, the health of residents can be greatly improved by increasing the medical level. In the CPL city cluster, the correlation between the medical level and the health of residents takes an inverted U shape, and the correlation curve has not reached the inflection point; i.e., the growing medical level has not effectively enhanced the health of residents. Overall, it is possible to suppress the negative impacts of environmental pollution and promote the health of residents by expanding the coverage and rationalizing the allocation of medical resources.
(4) For city clusters along the middle reaches of the Yangtze River, the correlation between environmental governance and the health of residents always exists as an inverted U. This means the city clusters have not invested enough in environmental protection to offset the negative impacts on the health of residents from environmental pollution.

In light of the above findings, the following suggestions were made to improve the health of residents in city clusters along the middle reaches of the Yangtze River:

(1) Economic development should be vigorously promoted, and the regional industrial structure should be optimized to find new drivers of economic growth. Economic growth is still an important basis for ensuring the health of residents. City clusters along the middle reaches of the Yangtze River should promote high-quality economic development, change the extensive development model, constantly optimize the internal economic structure, aim at new drivers of economic development and lay a solid material foundation for ensuring the health of residents.
(2) The local governments should increase fiscal investments on medical services, improve the social infrastructure and coordinate regional service resources. In addition to pursuing economic growth, the local governments should improve the livelihood security system, increase public medical expenditure and encourage social funds to enter the health field. The governments should also optimize the basic conditions of health, improve medical technologies, expand medical coverage and reduce the health burdens of residents. Moreover, the social service resources should be distributed more rationally between urban and rural areas, without sacrificing the service quality.
(3) Pollution emissions should be reduced. Green development should be pursued, and environmental governance should be deepened. Cities along the middle reaches of the Yangtze River should change their development concepts, constantly integrate with the ecological civilization, gradually ban enterprises with high pollution and high energy consumption, strengthen the management of pollutant discharge and waste from enterprises and reduce air and soil pollution. At the same time, alternative clean energy should be actively excavated to reduce energy intensity, and environmental pollution should be curbed at the source. On the basis of the environmental carrying capacity, environmental governance should be strengthened. Strict supervision mechanisms should be established, and pollution prevention and control should be vigorously promoted to improve the living environment.

There are still some deficiencies, which embodied the data acquisition and other reasons. Some variables, such as the level of economic development and the level of medical care, are not well-selected, which makes it difficult to reflect the actual situation of each city group in a more comprehensive and subtle way. Therefore, it may have a certain impact on the evaluation results. Future research work will further build a more accurate and complete indicator system, improve the evaluation and measurement methods and conduct an in-depth discussion on the significant results of regression, so as to obtain more objective and accurate results.

## Figures and Tables

**Table 1:** Statistical description of the variables. CZT: the Changsha-Zhuzhou-Xiangtan city cluster. CPL: the circum-Poyang Lake city cluster. health: the health of residents is measured by the mortality rate; lngdpr: the logarithmic form of the regional GDP growth rate; Inenvp: the logarithmic form of environmental pollution; Inhosp: the logarithmic form of the number of medical institutions; envm: the average rate of the municipal sewage treatment rate, the comprehensive utilization rate of industrial solid waste and the harmless treatment rate of household garbage.

**Variables**	**Unit**	**(CZT) City Cluster**				**Wuhan City Cluster**			
		**Mean**	**Standard Deviation**	**Max**	**Min**	**Mean**	**Standard Deviation**	**Max**	**Min**
health	One-thousand	7.10	0.41	8.45	6.37	5.12	1.24	7.91	2.50
lngdpr	percentage	2.48	0.20	2.77	2.03	2.47	0.24	2.80	1.67
lnenvp	Ten-thousand tons	13.70	0.91	15.73	10.99	13.69	1.18	15.45	10.05
lnhosp	one	5.31	0.38	5.99	4.64	4.94	0.55	6.19	3.61
envm	percentage	82.03	14.14	98.00	35.77	74.45	15.53	97.68	39.77
**Variables**	**Unit**	**(CPL) City Cluster**				**City Clusters Along the Middle Reaches of the Yangtze River**			
		**Mean**	**Standard Deviation**	**Max**	**Min**	**Mean**	**Standard Deviation**	**Max**	**Min**
health	One-thousand	6.03	0.18	6.33	5.34	6.08	1.11	8.45	2.50
lngdpgr	percentage	2.51	0.20	2.84	2.14	2.49	0.22	2.84	1.67
lnenvp	Ten-thousand tons	13.62	1.17	15.22	10.93	13.67	1.11	15.73	10.05
lnhosp	one	4.98	0.71	6.13	3.47	5.08	0.60	6.19	3.47
envm	percentage	79.11	16.50	99.50	22.84	78.53	15.81	99.50	22.84

**Table 2:** Results of the cointegration test. PP: Phillips-Perron and ADF: Augmented Dickey-Fuller.

Panels	Panel PP	Panel ADF	Group PP	Group ADF	Kao statistic
(CZT) City Cluster	−5.12 ***	−3.63 ***	−9.83 ***	−5.82 ***	−2.50 ***
Wuhan City Cluster	−7.81 ***	−4.73 ***	−14.36 ***	−6.65 ***	−2.59 ***
(CPL) City Cluster	−1.53 *	−1.58 *	−3.12 ***	−2.56 ***	−2.19 **
City Clusters Along the Middle Reaches of the Yangtze River	−8.88 ***	−6.16 ***	−15.70 ***	−8.62 ***	−3.96 ***

Note: ***, ** and * mean the significance at the 1%, 5% and 10% levels, respectively.

**Table 3:** Regression results on key explanatory variables. C: constant; R^2^: coefficient of determination.

Variables	CZT City Cluster		Wuhan City Cluster		CPL City Cluster		City Clusters Along the Middle Reaches of the Yangtze River	
	Model 1	Model 2	Model 3	Model 4	Model 5	Model 6	Model 7	Model 8
C	8.78 ***	8.11 ***	6.62 ***	4.50 ***	6.56 ***	5.72 ***	7.09 ***	6.12 ***
lngdpgr	−0.68 ***	−0.64 ***	−0.61 ***	−0.52 ***	−0.21 ***	−0.17 ***	−0.43 ***	−0.34 ***
lnenvp		0.04 ***		0.14 ***		0.05 ***		0.05 ***
F value	28.4 ***	25.9 ***	330.1 ***	228.1 ***	22.6 ***	61.8 ***	63.1 ***	62.1 ***
$R^{2}$	0.70	0.71	0.97	0.96	0.66	0.85	0.84	0.84
Sample size	104	104	130	130	130	130	364	364

Note: ***, ** and * mean the significance at the 1%, 5% and 10% levels, respectively.

**Table 4:** Regression results of our model for the health of residents. ln(envm)^2^: quadratic term of the logarithm of the environmental governance; ln(hosp)^2^: quadratic term of the logarithm of the medical level.

Variables	CZT City Cluster		Wuhan City Cluster		CPL City Cluster			City Clusters Along the Middle Reaches of the Yangtze River	
	Model 9	Model 10	Model 11	Model 12	Model 13	Model 14	Model 15	Model 16	Model 17
C	8.34 ***	12.87 ***	4.98 ***	13.88 ***	5.06 ***	5.87 ***	5.65 ***	5.85 ***	8.04 ***
lngdpgr	−0.56 ***	−0.58 ***	−0.54 ***	−0.52 ***	−0.19 ***	−0.18 ***	−0.18 ***	−0.30 ***	−0.30 ***
lnenvp	0.01	−0.01	0.11 ***	0.15 ***	0.06 ***	0.05 ***	0.05 ***	0.06 ***	0.05 ***
lnhosp	−0.07 **	−0.09 **	−0.07	−0.12	0.11 ***	0.12 ***	0.21 ***	0.007	−0.02
envm	0.01 ***	0.06 ***	0.38 *	0.13 ***	0.002 ***	0.01 ***	0.01 ***	0.001 *	0.03 ***
ln(envm)^2^		−0.46 ***		−0.10 ***		−0.10 ***			−0.25 ***
ln(hosp)^2^							−0.016 *		
F-value	19.3 ***	22.3 ***	163.9 ***	80.0 ***	101.3 ***	248.2 ***	97.3 ***	59.7 ***	63.2 ***
$R^{2}$	0.7	0.75	0.95	0.91	0.92	0.97	0.92	0.85	0.86
Sample size	104	104	130	130	130	130	130	364	364

Note: ***, ** and * mean the significance at the 1%, 5% and 10% levels, respectively.
